# Analytical Sensitivity Comparison between Singleplex Real-Time PCR and a Multiplex PCR Platform for Detecting Respiratory Viruses

**DOI:** 10.1371/journal.pone.0143164

**Published:** 2015-11-16

**Authors:** Jayme Parker, Nisha Fowler, Mary Louise Walmsley, Terri Schmidt, Jason Scharrer, James Kowaleski, Teresa Grimes, Shanann Hoyos, Jack Chen

**Affiliations:** 1 Department of Health and Social Services, Division of Public Health, Alaska State Public Health Virology Laboratory, Fairbanks, Alaska, United States of America; 2 Department of Biology and Wildlife, Institute of Arctic Biology, University of Alaska Fairbanks, Fairbanks, Alaska, United States of America; University Medicine Greifswald, GERMANY

## Abstract

Multiplex PCR methods are attractive to clinical laboratories wanting to broaden their detection of respiratory viral pathogens in clinical specimens. However, multiplexed assays must be well optimized to retain or improve upon the analytic sensitivity of their singleplex counterparts. In this experiment, the lower limit of detection (LOD) of singleplex real-time PCR assays targeting respiratory viruses is compared to an equivalent panel on a multiplex PCR platform, the GenMark eSensor RVP. LODs were measured for each singleplex real-time PCR assay and expressed as the lowest copy number detected 95–100% of the time, depending on the assay. The GenMark eSensor RVP LODs were obtained by converting the TCID_50_/mL concentrations reported in the package insert to copies/μL using qPCR. Analytical sensitivity between the two methods varied from 1.2–1280.8 copies/μL (0.08–3.11 log differences) for all 12 assays compared. Assays targeting influenza A/H3N2, influenza A/H1N1pdm09, influenza B, and human parainfluenza 1 and 2 were most comparable (1.2–8.4 copies/μL, <1 log difference). Largest differences in LOD were demonstrated for assays targeting adenovirus group E, respiratory syncytial virus subtype A, and a generic assay for all influenza A viruses regardless of subtype (319.4–1280.8 copies/μL, 2.50–3.11 log difference). The multiplex PCR platform, the GenMark eSensor RVP, demonstrated improved analytical sensitivity for detecting influenza A/H3 viruses, influenza B virus, human parainfluenza virus 2, and human rhinovirus (1.6–94.8 copies/μL, 0.20–1.98 logs). Broader detection of influenza A/H3 viruses was demonstrated by the GenMark eSensor RVP. The relationship between TCID_50_/mL concentrations and the corresponding copy number related to various ATCC cultures is also reported.

## Introduction

Multiplex PCR methods, those that target more than one pathogen in a single test, benefit diagnostics in a clinical laboratory due to their ability to detect and rule-out many related pathogens in the same amount of time. New and improved workflow designs make it possible for laboratories with varied molecular technical ability to implement multiplex PCR platforms.

The Respiratory Viral Panel (RVP) manufactured by GenMark Diagnostics, Inc. is a multiplex PCR panel that detects the amplification of various viral gene fragments electrochemically. Nucleic acids from targeted viral pathogens are amplified using a multiplex PCR reaction followed by denaturation of the double stranded molecules into single oligonucleotide strands using exonuclease. Once the amplicons are in a single-stranded state, they are hybridized to a complementary virus-specific signal probe tagged with ferrocene, a reducing agent. This hybridized molecule is then exposed to another sequence-specific probe which is bound to a solid phase, a gold electrode. Upon application of a low voltage current, the hybridized molecule bound to this solid phase brings the ferrocene in close proximity to the gold electrode where reversible electron transfer can occur and the resulting current can be measured. Viral pathogenic nucleic acid can be detected with confidence when measurements are at or exceed 3 nanoamps (nA) on the GenMark XT-8 instrument. The GenMark eSensor RVP has been shown to be highly comparable to other multiplex PCR platforms as well as singleplex real-time PCR in terms of diagnostic sensitivity and specificity[1,2], which measures the level of correlation between two methods. In this experiment, the primary interest is the analytical sensitivity of the PCR assays, or the minimum detectable concentration of the target. The GenMark eSensor RVP LODs as determined by the manufacturer are compared to singleplex real-time PCR assay LODs determined by our laboratory and expressed as lowest copy number reliably detected 95–100% of the time.

Limit of detections for FDA-approved clinical assays, including those described in the GenMark eSensor RVP package insert, are commonly expressed as 50% tissue culture infectious dose per milliliter, or TCID_50_/mL. Although this is a standard practice, other quantification methods such as real-time PCR are also reliable and may be able to more precisely describe quantities of viral particles with or without TCID_50_/mL calculations as a reference[3–6]. Since the LODs for the GenMark RVP assays are expressed exclusively as TCID_50_/mL concentrations, these values needed to be converted to copy number per μL in order to meet our goals of comparing analytical sensitivity as lowest copy number. The LODs of each GenMark RVP assay were not re-established in our laboratory. Instead, manufacturer established TCID_50_/mL values were converted to copy number using quantitative real-time PCR (qPCR). Performing this conversion also provided an opportunity to view the relationship between TCID_50_/mL and copy number and relate this information to various virus-infected ATCC cell cultures.

The respiratory assays evaluated in this experiment target the following virus species: influenza A virus (InfA/H3N2 and InfA/H1N1pdm09), influenza B virus (InfB), human respiratory syncytial virus (RSV), human parainfluenza virus (hPIV 1, 2, and 3), human adenovirus (Adeno), and human rhinovirus (hRV). The multiplex GenMark eSensor RVP assays were able to further distinguish human adenoviruses as belonging to subgenera C or E and respiratory syncytial viruses as belonging to subgroup A or B, unlike the singleplex real-time PCR assays that were designed to detect human adenovirus and respiratory syncytial virus universally across all subgroups. A generic influenza A virus assay, one that targets a conserved region of all influenza A viruses regardless of strain, was also evaluated.

## Methods and Materials

### Clinical specimens

Clinical specimens used in this study were de-identified. The University of Alaska Fairbanks Institutional Review Board (IRB) has determined that the proposed research qualifies for exemption from the requirements of 45 CFR 46 (Approval number: 667418–1).

### Preparation of standard materials

Specific plasmids were created for each real-time PCR assay by ligating single copies of the diagnostic amplicon onto vectors (pCR 2.1 or pCR4, Invitrogen) and amplifying via TOPO cloning (Invitrogen). Transformant *E*.*coli* competent cells were extracted using a phenol/chloroform mixture and the presence of viral-specific inserts was verified by sequencing (Elim Biopharmaceuticals, Inc.).

Plasmid concentrations were calculated by performing two quantification methods: 1) fluorometry specific to double stranded DNA (Qubit 2.0, dsDNA br Assay Kit, Invitrogen) and 2) pixel intensity measurements using the ImageJ application[7]. Using ImageJ, the pixel intensity of linearized plasmid DNA gel bands could be interpolated into a standard curve consisting of 1KB ladder dilutions (New England Biolaboratories) to predict quantities of unknown bands on the gel. Plasmid DNA was linearized using restriction enzyme NcoI (New England Biolabs) prior to gel electrophoresis. These quantification strategies were chosen to focus on the DNA of interest and to help exclude possible quantification pitfalls of over or underestimating DNA concentrations. Used in combination, these methods accounted for contaminating RNA (fluorometry specific for DNA only) as well as contaminating DNA as seen as different sized bands on the gel which could be excluded by only measuring the pixel intensity of gel bands of expected size (~4KB).

Differences between the two quantification methods ranged from 0.2 to 5.4 ng/μL (average 2.6 ng/μL ± 1.8). Final concentrations were calculated by rounding the average of the two methods to the nearest 2.5ng/μL. The weight of each plasmid was calculated using Geneious (v.8.1.3), using the known sequence of the vector in addition to the confirmed sequence of the insert. Final copy numbers (per μL) were calculated by dividing the plasmid weights (ng/copy) into the concentrations of each plasmid (ng/μL). Results of the quantification methods and downstream calculations are shown in Table 1.

**Table 1:** Plasmid concentrations and copy number determination.

Virus target insertion	Vector	Concentration (ng/μL)		avg ± SD (ng/ μL)	Final (ng/ μL)	Weight per plasmid copy* (ng)	Copies/μL
		*Qubit*	*ImageJ*				
Adeno	pCR2.1	8.0	7.8	7.9 ± 0.2	7.5	4.17 x 10^−9^	1.80 x 10^9^
InfA	pCR 2.1	10.1	11.5	10.8 ± 1.0	10	4.14 x 10^−9^	2.42 x 10^9^
InfA/H3N2	pCR4	14.5	9.1	11.8 ± 3.8	10	4.37 x 10^−9^	2.29 x 10^9^
InfA/H1N1pdm09	pCR2.1	11.1	11.8	11.5 ± 0.5	10	4.15 x 10^−9^	2.41 x 10^9^
InfB	pCR4	26.0	31.1	28.6 ± 3.6	30	4.17 x 10^−9^	7.19 x 10^9^
hPIV-1	pCR4	13.2	8.7	9.5 ± 2.0	10	4.32 x 10^−9^	2.31 x 10^9^
hPIV-2	pCR4	7.4	6.7	7.0 ± 0.5	7.5	4.15 x 10^−9^	1.81 x 10^9^
hPIV-3	pCR4	38.8	35.0	36.9 ± 2.7	35	4.19 x 10^−9^	8.35 x 10^9^
RSV	pCR4	5.1	6.2	5.7 ± 0.8	5	4.17 x 10^−9^	1.20 x 10^9^
hRV	pCR4	32.7	29.8	31.3 ± 2.1	30	4.27 x 10^−9^	7.03 x 10^9^

*Weight/copy was calculated using Geneious (v.8.1.3) which considers the exact sequence of the plasmid.

### Determination of singleplex real-time LOD

Plasmid DNA was serially diluted to produce eight (8) test concentrations ranging between 1 copies/μL and 1250 copies/μL, depending on the assay. This narrow range was chosen to identify the lowest potential copy number able to be detected repeatedly, but keep it above theoretical limitations of real time PCR, <3 copies (0.6 copies/uL when using 5uL per reaction)[8]. Seven (7) replicates were tested at each concentration. This process was repeated twice, once using nuclease-free water as the diluent background for the plasmids to assess basic analytical sensitivity and once using total nucleic acid extract (TNA) as background for the plasmids to simulate real clinical matrices. TNA was isolated from clinical specimens using the easyMAG total nucleic acid automated extractor (Biomerieux). A total of 200μL of the clinical specimen was extracted and final eluate volumes were 60μL. TNA from clinical specimens were screened by PCR, and only those that demonstrated the absence of target DNA or RNA were qualified to be pooled as clinical background diluent.

Primers and probes used in the laboratory-developed real-time PCR assays have been previously described[9,10]. Influenza assays were performed using Invitrogen Superscript III reagents and all other assays were performed using Ambion AgPath ID reagents. For assays using the Invitrogen reagents, the following PCR thermal cycling profile was used; 50°C hold for 30 minutes, 95°C hold for 2 minutes, and 45 cycles of 95°C for 15 seconds then 55°C for 30 seconds. For assays using the Ambion reagents, the following PCR thermal cycling profile was used; 45°C hold for 10 minutes, 95°C for 10 minutes, and 45 cycles of 95°C for 15 seconds then 55°C for 1 minute. Reactions were tested using ABI 7500Dx thermal cyclers (Life Technologies).

Negative controls consisted of no template control replicates (NTC, n = 3) and diluent blank replicates, made up of water or TNA diluent (n = 7) to assess contamination. Positive reactions were defined as those amplification curves that produced cycle threshold (Ct) values at or below 40 cycles. The LOD was chosen as the concentration that demonstrated a percentage of positivity over all replicates at a particular dilution. The percentage of positivity was chosen using those that were set by the manufacturer for each matching GenMark RVP assay. All but three assays were set by the manufacturer below 100% positivity (InfA/H1N1pdm09, RSVA, and hRV assays only); therefore, the LOD for these particular singleplex assays were estimated using probit analysis to match these probabilities for comparison purposes[11]. Final LODs were expressed as a concentration, copies/μL (Table 2).

**Table 2:** LOD comparison summary.

		Singleplex Real-time PCR		Multiplex PCR GenMark eSensor RVP	copies/μL difference	Log Difference
		Lowest copies/μL detected		copies/μL equivalent of TCID_50_/mL LOD		
Assay	%pos	*Clinical background*	*No Background*			
**Adeno C**	100%	1.6	4	110.4 ± 8	108.8	2.04
**Adeno E**	100%	1.6	4	390.4 ± 45.4	388.8	2.59
**InfA**	100%	5.4	21.2	1286.2 ± 23.2	1280.8	3.11
**InfA/H3N2**	100%	10.6	21.2	<2.2	8.4	0.92*
**InfA/H1N1**	97.5%	7	7	10 ± 4.4	3	0.48
**InfB**	100%	2.6	53.2	1 ± 2.8	1.6	0.20*
**hPIV-1**	100%	1	1	<2.2	1.2	0.08
**hPIV-2**	100%	5.4	2.2	1.6 ± 0.6	3.8	0.58*
**hPIV-3**	100%	2.2	1	134.8 ± 8.4	132.6	2.12
**RSVA**	97.5%	6.8	3.6	326.2 ± 22.8	319.4	2.50
**RSVB**	100%	10.6	5.2	120.2 ± 8.6	109.6	2.04
**hRV**	95%	111.8	82.4	<17	94.8	1.98*

*lower LOD demonstrated for the multiplex assay; 5μL used in each reaction. Adenovirus and RSV assays were not differentiated with the singleplex real-time PCR assay, although RSV assays were calculated differently based on %pos to be compared. The TCID_50_/mL concentration for InfA/H3, HPIV 1, and hRV exceeded the detection limit on the qPCR assay. Copy number difference was calculated by subtracting the lowest copies/μL detected with clinical background on the singleplex assays from the average copies/μL equivalent converted from TCID_50_/mL.

### Conversion of TCID50/mL concentrations to copies/μL

Cell cultures with known TCID_50_/mL quantities of target viruses (ATCC) were used to estimate the LOD for the GenMark RVP assay. Cultures were stored in liquid nitrogen until they were extracted using the easyMAG total nucleic acid automated extractor (Biomerieux). A total of 200μL of the TCID_50_/mL culture was extracted and final eluate volumes were 60μL. Purified nucleic acid was stored at -80°C until tested by quantitative real time PCR (qPCR).

Using quantified plasmids containing inserts specific to each assay, ten-fold dilutions were prepared covering 10^1^ to 10^6^ copies/5μL. Each dilution was tested in triplicate to create a standard curve. All qPCR assays utilized a sequence-specific hydrolysis probe with the exception of the H3 due to sequence incompatibilities with the ATCC strain being analyzed (see results). In this case, a SYBR Green assay (GoTaq, Promega) with new primers were designed to target this specific strain of Influenza A/H3. Alongside the standard curve, dilutions of the isolated nucleic acid derived from the ATCC cultures were tested in triplicate at dilutions that would include reported GenMark eSensor RVP LOD TCID_50_/mL values. As with the singleplex real-time PCR assays, reactions were tested on ABI 7500Dx thermal cyclers (Life Technologies) and standard curves and associated unknown quantities were calculated using ABI 7500 v2.3 software. The copy number equivalents for each GenMark eSensor RVP assay’s LOD is shown in Table 2. The relationship between copy number and TCID_50_/mL for each ATCC culture tested is shown in Table 3.

**Table 3:** Relationship between TCID50/mL concentrations and copy number.

ATCC Culture		Genome Copies/TCID_50_ (± SD)	LOD for GenMark eSensor RVP (TCID_50_/mL)
VR-1	Adenovirus serotype 1 (subgenera C)	7 ± 1	8.89 x 10^1^
VR-1572	Adenovirus serotype 4 (subgenera E)	124 ± 14	1.58 x 10^1^
VR-547	Influenza A/H3 (Aichi)	0.01 ± 0	1.58 x 10^3^
VR-1736	Influenza A/H1N1	2,381 ± 1,048	1.05 x 10^−1^
VR-101	Influenza B	16 ± 44	3.16 x 10^−1^
VR-94	Human Parainfluenza Virus 1 (C35)	391 ± 0	2.81 x 10^−2^
VR-92	Human Parainfluenza Virus 2 (Greer)	0.03 ± 0.01	2.81 x 10^2^
VR-93	Human Parainfluenza Virus 3 (C243)	24 ± 1	2.81 x 10^1^
VR-1540	Respiratory Syncytial Virus (A2)	6 ± 1	2.81 x 10^2^
VR-955	Respiratory Syncytial Virus (B9320)	38 ± 3	1.58 x 10^1^
VR-483	Rhinovirus 3 FEB	53,797 ± 0	1.58 x 10^−3^

SD = standard deviation, SD could not be calculated for VR-547, VR-94, and VR-483 since the TCID_50_/mL concentration exceeded the detection limit on the qPCR assay.

## Results

Ten singleplex real-time PCR assays were compared in terms of analytical sensitivity to twelve multiplex assays on the GenMark eSensor RVP. This difference stems from the fact that the singleplex real-time PCR assays are not designed to distinguish between different subgenera of human adenovirus or different subtypes of respiratory syncytial viruses (RSV), while the GenMark eSensor RVP differentiates between human adenovirus C and E as well as RSV subtype A and B. Thus, two additional assays were evaluated for the GenMark eSensor RVP. Analytical sensitivity was expressed as lowest copies/μL concentration for all assays.

The Genmark eSensor RVP capable of distinguishing between different subgenera of adenoviruses (C vs. E) demonstrated less analytical sensitivity than the generic singleplex real-time PCR assay targeting all adenoviruses, differing by 108.8 copies/μL (2.04 log difference), and 388.8 copies/μL (2.59 log difference), respectively. The difference in sensitivity may be due to slight variations in the targeted priming region. The singleplex real-time PCR assays use primers designed to anneal highly conserved sequences within the hexon-coding region in order to target all adenoviruses, whereas the GenMark eSensor RVP assays use subgenera-specific hexon primers to make possible the distinction between adenovirus subgenera C and E. Upper respiratory tract infections associated with adenovirus C viruses infect more than 80% of the population early in life[12]; however, infections with the adenovirus E (serotype 4) can prove to be more severe and even fatal for people living in close quarters, such as military recruits[13]. In terms of surveillance, differentiation of virus subgenera within a population may be clinically useful, regardless of lost sensitivity.

Similarly, the singleplex real-time PCR assay generically targeting respiratory syncytial viruses also demonstrated better sensitivity than the GenMark eSensor RVP assays which are capable of distinguishing between subtypes A and B (319.4 copies/μL, 2.50 log difference and 109.6 copies/μL, 2.04 log difference, respectively). Respiratory syncytial viruses in subtype A are thought to be more prevalent and virulent than those in subtype B[14]. Subtyping respiratory syncytial virus may be clinically beneficial when surveilling populations that experience high hospitalization rates associated with the virus, such as Native Americans living in southwest United States and Alaska[15].

Analytical sensitivity of assays targeting the current circulating strains of influenza A viruses in the human population, H3N2 and H1N1pdm09, were highly comparable between the singleplex real-time PCR and multiplex GenMark eSensor RVP assays (8.4 copies/μL, 0.92 log difference and 3 copies/μL, 0.48 log difference, respectively). Comparing the LOD between the influenza H3N2 assays proved to be the most challenging. When converting TCID_50_/mL concentrations to copies/μL using qPCR, it was determined that this particular culture contained an uncommon virus, an Aichi strain (A/Aichi/2/35) circa 1968 (ATCC) and therefore could not be amplified using the singleplex real-time PCR assay, which is designed to detect current influenza A/H3N2 virus strains. However, it was repeatedly detected using the GenMark eSensor RVP. This finding suggests that the eSensor RVP is capable of detecting a broader range of Influenza A/H3N2 strains while maintaining a comparable analytic sensitivity to that of its singleplex real-time PCR counterpart.

The greatest difference measured between analytic sensitivities was seen with the generic influenza A assay showing a 3.11 log difference in LOD (1280.8 copies/μL difference). Because the LOD for the generic influenza A assay is much higher than the subtype assays (as described above) for the multiplex GenMark eSensor RVP, difficulty in result interpretation from specimens with low influenza A virus titers is likely, since subtypes (H3N2 or H1N1pdm09) have a lower LOD than the generic influenza A assay (e.g. + H3N2,—influenza A). The performance of the generic influenza A assay is an important surveillance tool for tracking genetic changes among influenza A viruses. For instance, specimens demonstrating positivity for influenza A using this generic, highly conserved matrix-coding region may not subtype using the H3N2 or H1N1pdm09 assays, which may indicate that the virus is novel and worthy of alerting public health authorities. In contrast, the influenza B assays were shown to be highly comparable between the singleplex and multiplex assays, with a difference of only 1.6 copies/μL (0.20 log difference).

Human parainfluenza 1 assays were highly comparable (1.2 copies/μL, 0.08 log difference). Human parainfluenza 2 assays demonstrated improved sensitivity on the multiplex GenMark eSensor assay (3.8 copies/μL, 0.58 log difference). Human parainfluenza 3 assays demonstrated the largest difference in analytical sensitivity among the human parainfluenza serotypes, demonstrating a 2.12 log improvement in detectability when using the singleplex real-time PCR assay (132.6 copies/μL difference).

Five of the twelve GenMark eSensor RVP assays matched (<1 log difference in copies/μL) the LOD of the real-time singleplex PCR assay targets in this study (Table 2). These include influenza A/H3N2, influenza A/H1N1pdm09, influenza B, and human parainfluenza 1 and 2. Six of the twelve assays compared showed greater sensitivity using the real-time singleplex assays. These include the adenovirus assays (C & E), influenza A, human parainfluenza 3, and RSV (A & B). The GenMark eSensor human rhinovirus assay demonstrated the biggest difference in terms of improved detection when compared to its singleplex counterpart (94.8 copies/μL, 1.98 log difference, 95% positivity).

The number of genome copies per TCID_50_/mL value was highly variable ranging from 0.01 to 53,797 (Table 3). LODs set at higher TCID_50_/mL concentrations (10^2^–10^3^) corresponded to stock cultures with lower copy numbers (0.01 to 6 copies). LODs set at in the mid-range TCID_50_/mL concentrations (10^1^ to 10^−1^) corresponded to stock cultures with variable copy numbers per TCID_50_/mL (7–2,381 copies). LODs set at lower TCID_50_/mL concentrations (10^−2^–10^−3^) corresponded to stock cultures with somewhat higher copy numbers per TCID_50_/mL (391–53,797 copies).

## Conclusion

Multiplex PCR applications benefit diagnostics in a clinical laboratory due to their ability to detect and rule-out many related pathogens in a single reaction, reducing tech-time by more than 3 hours for a panel of 10 viruses[1]. However, multiplex PCR platforms continue to carry higher overall costs. Analytic sensitivity, or the lowest possible concentration necessary to produce a reliable result, is an important parameter to consider when replacing singleplex real-time PCR assays with multiplex PCR platforms evolving from newer, more expensive technologies. This experiment aims at finding a method in which to compare LODs of various assays using copy number as the unit of expression.

Choosing a 2.5 log difference to express considerable loss in sensitivity, the multiplex PCR strategy in combination with the GenMark eSensor technology demonstrates a considerable loss in sensitivity for three of the twelve assays assessed. Two of the assays were adenovirus E and respiratory syncytial virus subtype A. Although sensitivity is reduced, further characterization of viruses in clinical specimens may be of greater clinical importance, especially when particular subtypes are known to be more virulent in the population as is the case with adenovirus serotype 4 (subgenera E) and respiratory syncytial virus subtype A in particular populations.

The third assay demonstrating considerable loss in sensitivity was for the generic influenza A assay. Clinical laboratories, especially those directly related to public health surveillance, may need to consider the significance of this reduced sensitivity since it is commonly used to rule out novel influenza. Better analytic sensitivity was achieved using singleplex real-time PCR, which indicates that influenza A can be detected in clinical specimens even at low titers using this method. Specimens collected from patients that are suspected to have influenza infections that test negative on the GenMark eSensor RVP may need to be tested by more sensitive methods to rule out cases of novel influenza.

Expressing LOD in units that can be comparable across methodologies can prove to be difficult experimentally. TCID_50_/mL measurements can vary depending on how these cultures are handled in the laboratory in regards to preserving the concentration of infectious virus particles for purposes of experimentation and quantity comparisons. Molecular detection strategies used in clinical laboratories are non-discriminating when identifying infectious or non-infectious viruses. PCR methodologies used to detect viral targets in clinical specimens do not provide information regarding the viability of the virus and, therefore, every detection may not point to a causative agent of disease. Other complicating factors to consider when interpreting PCR results are that patients can be asymptomatic carriers or may be exhibiting evidence of a past infections. Viral copy numbers provide an estimate of the number of virus particles in a given volume, but in our experiment, they did not correlate well with the number of infectious particles. To test the analytical sensitivity of a PCR-based methodology, it is important to understand that the intent of the assay is to detect any genome copy targeted by the designed primers, whether these be from infectious or non-infectious virus particles.
