# Correction: Analytical Sensitivity Comparison between Singleplex Real-Time PCR and a Multiplex PCR Platform for Detecting Respiratory Viruses

**DOI:** 10.1371/journal.pone.0205483

**Published:** 2018-10-04

**Authors:** Jayme Parker, Nisha Fowler, Mary Louise Walmsley, Terri Schmidt, Jason Scharrer, James Kowaleski, Teresa Grimes, Shanann Hoyos, Jack Chen

There are errors in Table 3. The LOD reported for VR-92, VR-1540, and VR-955 is incorrect. Please see the corrected Table 3 here.

**Table 3:** Relationship between TCID50/mL concentrations and copy number.

ATCC Culture		Genome Copies/TCID_50_ (± SD)	LOD for GenMark eSensor assays (TCID_50_/mL)
VR-1	Adenovirus Type 1 (C)	7 ± 1	8.89 x 10^1^
VR-1572	Adenovirus Type 4 (E)	124 ± 14	1.58 x 10^1^
VR-547	Influenza A/H3 (Aichi)	0.01 ± 0	1.58 x 10^3^
VR-1736	Influenza A/H1N1	2,381 ± 1,048	1.05 x 10^−1^
VR-101	Influenza B	16 ± 44	3.16 x 10^−1^
VR-94	Human Parainfluenza Virus Type 1 (C35)	391 ± 0	2.81 x 10^−2^
VR-92	Human Parainfluenza Virus Type 2 (Greer)	0.03 ± 0.01	2.81 x 10^0^
VR-93	Human Parainfluenza Virus Type 3 (C243)	24 ± 1	2.81 x 10^1^
VR-1540	Respiratory Syncytial Virus (A2)	6 ± 1	2.81 x 10^0^
VR-955	Respiratory Syncytial Virus (B9320)	38 ± 3	1.58 x 10^0^
VR-483	Rhinovirus 3 FEB	53,797 ± 0	1.58 x 10^−3^

SD = standard deviation, SD could not be calculated for VR-547, VR-94, and VR-483 since the TCID_50_/mL concentration exceeded the detection limit on the qPCR assay.
